# Effect of Low Intensity Transcranial Ultrasound (LITUS) on Post-traumatic Brain Edema in Rats: Evaluation by Isotropic 3-Dimensional T2 and Multi-TE T2 Weighted MRI

**DOI:** 10.3389/fneur.2020.578638

**Published:** 2020-11-12

**Authors:** Tao Zheng, Juan Du, Yi Yuan, Shuo Wu, Yinglan Jin, Qinglei Shi, Xiaohan Wang, Lanxiang Liu

**Affiliations:** ^1^Department of Magnetic Resonance Imaging, Qinhuangdao Municipal No. 1 Hospital, Qinhuangdao, China; ^2^Institute of Electrical Engineering, Yanshan University, Qinhuangdao, China; ^3^Peking University Health Science Center, Beijing, China; ^4^Scientific Clinical Specialist, Siemens Ltd., Beijing, China

**Keywords:** LITUS, brain edema, ultrasound, therapy, neuroprotective

## Abstract

**Background:** Brain edema is one of the important factors affecting the prognosis of traumatic brain injury (TBI). Low-intensity transcranial ultrasound (LITUS) has significant anti-cerebral edema effect. T2-weighted image-based volume and T2 value measurements can sensitively reflect tissue edema.

**Purpose:** To evaluate the effect and possible mechanisms of LITUS on brain edema by iso-voxel 3-dimensional T2WI (iso-3D T2WI) and multi-TE T2WI. Methods: Forty-five rats were randomly divided into sham control, TBI and TBI + LITUS groups (*n* = 15, respectively). Iso-voxel 3-dimensional T2WI and multi-TE T2WI sequences at 3.0T to obtain T2 value and edema volume of the injury cortex. T2 values were obtained on days 1, 7, 14, 21, 28, 35, and 42 after TBI and brain edema volume were obtained on days 7 and 14.

**Results:** The T2 values of the damaged cortex in the TBI group showed a slow decreasing trend after a significant increase. For TBI+LITUS group, T2 values decreased with continuous LITUS treatment. At day 28, the T2 values were not significantly longer than the control group (adjusted *P* = 0.0535), but were significantly shorter than the TBI group at day 42 (adjusted *P* = 0.0003). The edema volume at day 7 and 14 in the LITUS group was significantly lower than the TBI group (*P* = 0.0004 and *P* < 0.0001, respectively). AQP-4 and β-APP protein staining showed a strong positive reaction near the CCI point, TBI+LITUS group showed a medium positive reaction, and the sham control group showed a weak positive reaction.

**Conclusion:** The therapeutic effect of LITUS on post-traumatic brain edema was confirmed through T2 value and edema volume, and the mechanism may be related to inhibiting the expression of AQP-4 and promoting the removal of β-APP.

## Introduction

Although it accounts for only 10–15% of total trauma, traumatic brain injury (TBI), as a common neurosurgical disease, has a far higher mortality rate than the injuries of other body parts, accounting for 85% of the total trauma death rate (1). While the majority of patients with mild-to-moderate TBI undergo clinical recovery, follow-up results show that 7–33% of the patients suffer from permanent Post-Concussion Syndrome (PCS) accompanied with physical, emotional, and cognitive injuries (2). Cerebral edema is the main factor that affects the mortality rate and functional recovery of cerebral trauma (3). A recent study found that TBI prognosis is significantly correlated with early common cerebral edema (4). The current research focus is to inhibit the occurrence of brain edema and rapidly induce edema regression after a traumatic injury. Drugs are currently the main course of clinical treatment; however, some have their efficacy limited by the blood-brain barrier, while other drugs such as mannitol often result in serious adverse reactions due to liver and kidney toxicity (5, 6). We currently lack a rapid and specific drug therapy for traumatic brain edema.

In the last 30 years, several breakthrough physiotherapy technologies have been added to neuromodulation methods, such as repetitive transcranial magnetic stimulation (rTMS), transcranial direct current stimulation (tDCS), optical stimulation and infrared stimulation. In comparison, low-intensity transcranial ultrasound stimulation (LITUS) is a technical method that penetrates the complete brain skull with low-intensity ultrasounds in order to modulate nerve function, with its characteristics being non-invasive, high spatial resolution and high stimulation depth (7). It has been widely used in the treatment of cerebrovascular embolism, Parkinson's disease (PD), Alzheimer's disease (AD), epilepsy, and tumor (8–11). LITUS alone can stimulate neural activity and increase the expression of neurotrophic factor (12). Previous studies using histological staining also found that LITUS can antagonize neuronal apoptosis and promote axonal regeneration in mice (13, 14). Our recent diffusion tensor imaging (DTI) and diffusion kurtosis imaging (DKI) studies have found that LITUS can inhibit the changes of functional parameters after TBI, which are closely related to neuronal injury, myelin sheath loss, gliosis and other pathological processes after TBI (15, 16). Recent research found that LITUS treatment can narrow the range of brain edema in an acute period of brain trauma and reduce the water content in the brain (13, 17). This suggests that LITUS may be a potential tool for the treatment of brain trauma.

Previous evaluation of edema degree after TBI primarily used clinical sign examinations and cerebrospinal fluid pressure measurements, the former having a high false negative rate and the latter being invasive. The development of magnetic resonance technology has provided a new way to evaluate cerebral edema. Currently, T2-Weighted imaging (T2WI) is used mainly to evaluate the efficacy and prognosis of post-traumatic cerebral edema. The degree of edema is reflected by the display of the scope of cerebral edema and measuring its volume. Based on T2 imaging technology, T2 mapping is also a quantitative magnetic resonance technique for the tissue T2 value, which describes specific transverse relaxation time of tissues and organs and evaluates their composition and functional changes. Many studies have confirmed that changes in tissue edema can occur during the early stage of lesions such as injury, ischemia, inflammation, and tumor. During this period, it is difficult to detect lesions using conventional MR technology due to the slight pathological changes. Fast spin-echo (FSE) T2WI is one of the most used T2WI sequences in clinical evaluation of traumatic brain edema, which can clearly show the location and extent of brain edema. Furthermore, compared with conventional FSE-T2WI, the image layer of 3D-space sequence is thinner, and the isotropic images with the same length, width and height of voxels can be obtained. The influence of partial volume effect can be avoided in the measurement of lesions, and the measurement results are more accurate. This study used iso-voxel 3-dimensional (3D) sampling perfection with application-optimized Contrast using different flip angle evolutions (SPACE) T2WI and multi-TE fast spin-echo (FSE) T2WI sequences to measure the volume and T2 value of damaged brain tissue, evaluated the effect of LITUS stimulation on post-traumatic brain edema, and explored the possible mechanism of LITUS on brain edema from macroscopic imaging's perspective. We also investigated protein molecular measurements in order to explore the possible mechanisms of treatment.

## Materials and Methods

### Rats

Sixty male Sprague–Dawley rats with weight of 250 ± 20 g and an age of 90 ± 10 days were randomly divided into three groups (see below). Each rat was raised in a separate cage. Animals were housed at 20–22°C, in 60% air humidity, and with food and water readily available. All rats were given an intraperitoneal injection with 10% chloral hydrate (4 mL/kg) prior to surgery and MR scan. In order to minimize the number of animals, this study follows the 3R principle of experimental animals. The experiment was approved by the Medical Ethics Committee and Animal Care. The animals were divided into three groups of 20 rats each. Five of them were taken at 1 day after the first LITUS stimulation and brain water content was measured. The remaining 15 rats underwent continuous MR and neurological behavioral scoring, of which 10 underwent pathological examination and 5 underwent brain water content measurements. Exclusion criteria were as follows: (a) Dura mater was damaged during Controlled Cortical Impact (CCI) operation in rats. (b) The rats died before the end of the study. (c) Head movement occurred during CCI of the rats, resulting in inaccurate damaged position.

Rats in the TBI group (*n* = 15) received a Controlled Cortical Impact (CCI) operation at beginning of the study. The model of moderate TBI in rats was established using a Feeney free fall device (eCCI Model 6.3; Custom Design, Richmond, VA, USA). The parameters of the impact device are as follows: the impact hammer weight was 21 g, the impact pipe length was 26 cm, impact bar diameter was 4.6 mm, and the end was 4 mm beyond the lower edge of the catheter. Freefall blows with these parameters can cause a moderate TBI (6). Rats were anesthetized by intraperitoneal injection with 10% chloral hydrate 4 mL/Kg and kept warm by light. The rat head was fixed on a plate, hair cut off from the top of the head and skin disinfected with 75% alcohol. An incision of about 1.5–2 cm was made in the middle of the parietal line. The skin was removed, parietal bone exposed and the right parietal periosteum peeled off. A bone window with a diameter of about 5 mm was ground to keep the dura intact, with the center 2.5 mm in the right of the midline and 1.5 mm behind the anterior fontanelle. After sterilizing the battering device, the dura was flushed with the lower end of the catheter and stuck to the impact rod. The catheter was ensured to vertical and the position of the brain of the rat not skewed. The impact hammer fell freely. The hammer was immediately lifted after impact. If bleeding occurred, a cotton ball was gently pressed to stop bleeding. The surgical field was cleaned, and the incision was sutured intermittently. The rats were first transferred to a thermal pad and then returned to the cage after a confirmation of physical activity. Rats in LITUS group (*n* = 15) received the same operation to establish moderate TBI model. After the CCI operation, LITUS was administered immediately to the rats and the rats were treated with LITUS stimulation once per day for 6 weeks (see below). During the stimulation, the rats were fixed on a stereotaxic apparatus to ensure the accuracy of each stimulus position. Rats in sham control group (*n* = 15) received a scalp incision and cranial drilling without a CCI operation.

### LITUS Protocol

**Figure 1:**
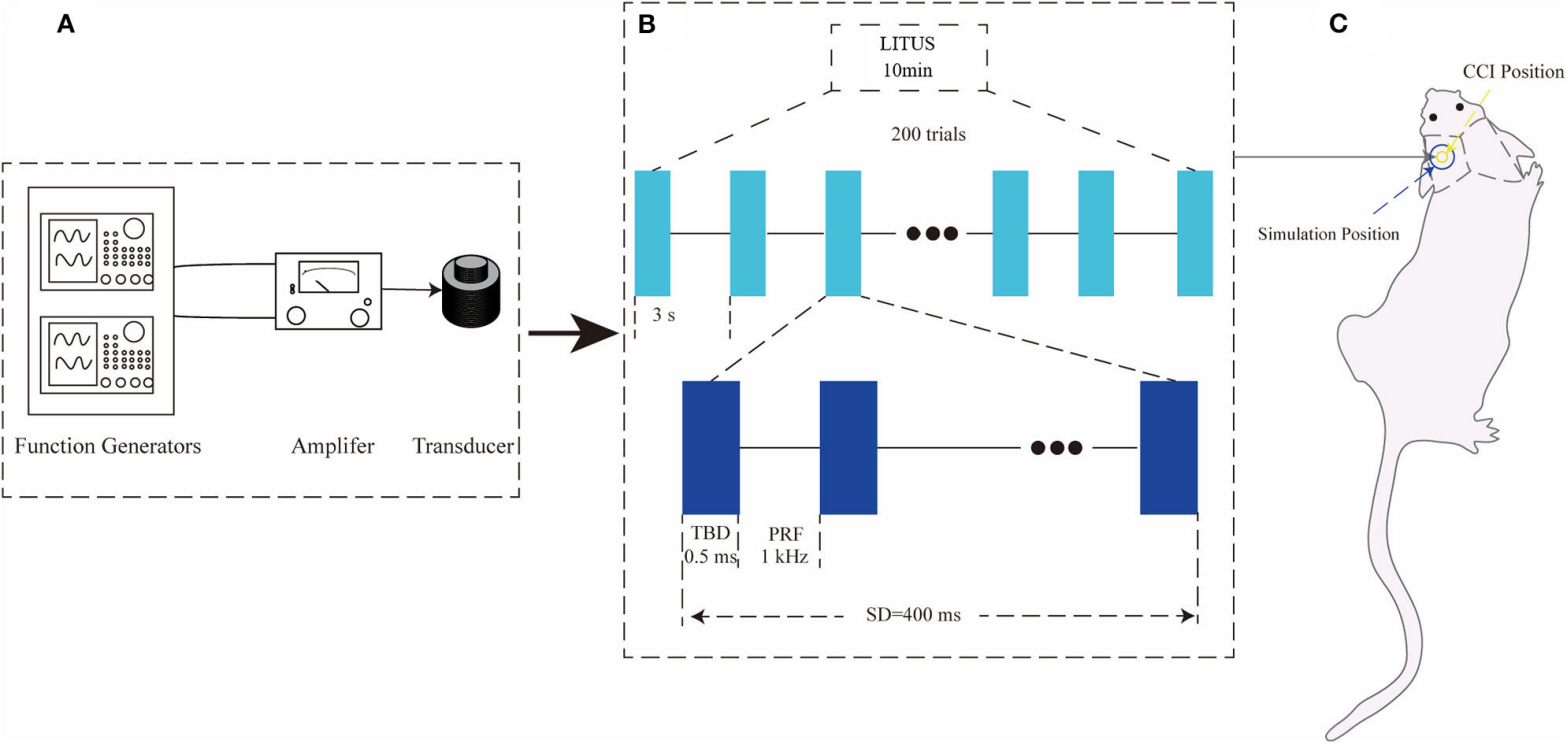
**(A)** LITUS system, two connected function generators were used to generate pulsed signals. The pulsed signal from the second generator was amplified by a linear power amplifier and transmitted to an unfocused ultrasound transducer. **(B)** The time schedule of ultrasound stimulation and the parameters of the ultrasound. The total stimulation duration was 10 min with 200 trials. The ultrasound FF and PRF were 500 and 1 kHz, respectively. The ultrasound SD and TBD were 400 and 0.5 ms, and the I_sppa_ value was 2.6 W/cm^2^ the time schedule of each group. **(C)** Ultrasonic stimulation was applied to the local area of CCI with a diameter of about 10 mm.

The ultrasonic stimulation system and parameters are the same as our previous studies (18). In the LITUS system, two generators (AFG3022C; Tektronix, USA) were used to generate the pulse signal. After amplification by a linear power amplifier (E&I240 L; ENI Inc., USA), the pulse signal is transmitted to the unfocused ultrasonic transducer (V301-SU; Olympus, Japan). After collimating through a 10 mm diameter collimator, ultrasound was applied to the damaged area of the rat (Figure 1A). Figure 1B shows the time and intensity parameters of ultrasound. The total stimulation duration was 10 min with 200 trials and the duration of each trial is 3 s. The ultrasound fundamental frequency (FF) and pulsed repetition frequency (PRF) were 500 kHz and 1 kHz, respectively. The tone-burst duration (TBD) and stimulation duration (SD) were 0.5 ms and 400 ms, respectively. The Isppa value was 2.6 W/cm^2^ (Figure 1B). During LITUS stimulation, the Non-invasive Vital Signs Monitor (V1.0, Shanghai Yuyan Instruments Co., Ltd.) was applied to monitor heart rate, respiration and blood pressure of rats to reflect humanistic care. Prior to each LITUS stimulation, anesthesia was performed by intraperitoneal injection of 10% chloral hydrate (4 mL/kg). During the stimulation, the rats were fixed on a stereotaxic apparatus to ensure the accuracy of each stimulus position (Figure 1C).

### MR Image Acquisition and Post-processing

The rats were scanned using a 3.0-Tesla Siemens TIM Verio Scanner (Siemens Medical Solutions, Erlangen, Germany) at days 1, 7, 14, 21, 28, 35, and 42 after TBI. The special 4-channel high-resolution animal coil (Shenzhen Super Electric Co., LTD, part no. 10-F04885) with a diameter of 50 mm was used for image acquisition. Each rat of the three groups was anesthetized with chloral hydrate before scanning. Anesthetic doses in each group were given as 4 mL/kg. Before anesthetics were administered, we measured each animal's weight, that is, the dosage of anesthetics was strictly controlled according to the body weight. After anesthesia, the rat head was immobilized using a custom-constructed MRI-compatible rat head holder during MR scanning. The image quality was analyzed after the MR scanning and if the rat's head had moved during the scanning, the scan was repeated 1 h later with additional anesthetics. To correct for gradient non-linearity effects and bias field on the measurement, images were corrected with the 2D or 3D distortion correction available on Statistical Parametric Mapping (SPM8).

The detailed parameters of MR sequences are as follows:

For iso-voxel 3D SPACE T2WI, the MR parameters were: Scan layers aligned perpendicular to the anterior/posterior line with the following settings: TR = 1,200 ms, TE = 113 ms, average = 6, FOV=64 × 64 mm, voxel size = 0.3 × 0.3 × 0.3 mm, data matrix=192 × 192, slice thickness = 0.3 mm, number of slices = 96 (total scanning time=8 min 18 s).

For multiple-TE FSE T2WI, the MR parameters were: Scan layers aligned perpendicular to the anterior/posterior line with the following settings: TR = 2,000 ms, TE 1 = 64 ms, TE 2 = 96 ms, TE 3 = 181 ms, flip angle = 180°, average = 6, FOV = 64 × 64 mm, voxel size = 0.3 × 0.3 × 2.0 mm, data matrix = 192 × 192, slice thickness = 2.0 mm, number of slices = 10 (total scanning time = 3 min 50 s).

For fast low-angle shot 2D T2WI (FLASH 2D T2WI), the MR parameters were: Scan layers aligned perpendicular to the anterior/posterior line with the following settings: TR = 4,000 ms, TE = 15 ms, flip angle = 15°, average = 6, FOV = 64 × 64 mm, voxel size = 0.3 × 0.3 × 2.0 mm, data matrix = 192 × 192, slice thickness = 2.0 mm, number of slices = 10 (total scanning time=3 min 50 s).

**Figure 2:**
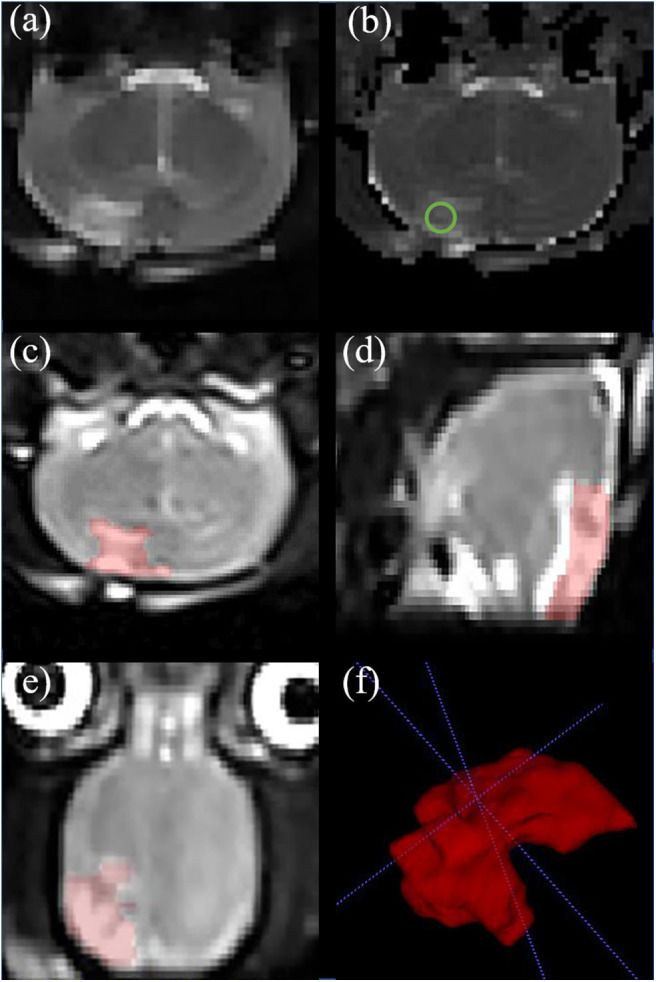
Representative rat brain images of a same rat in TBI group at day 7. **(a)** Coronal T2-weighted image. **(b)** T2 map. ROI of injury area (green circle in **b**) was chosen on an axial T2 map. **(c–e)** Iso-voxel 3D T2-weighted image. The red areas represent areas of edema in the brain. Through the application of Paintbrush Mode in ITK-SNAP, the volume of brain edema was obtained. **(f)** Three—dimensional morphology of cerebral edema after TBI.

T2 map was obtained by linear fitting of the natural logarithm of the signal intensity vs. TE using Image J software (NIH, Bethesda, Maryland, USA). The region of interest (ROI) range of 0.30–0.60 cm^2^ was manually selected in the center of the damaged cortex to measure the T2 value of brain tissue. ITK-SNAP software (Version 3.6.0) was used to manually delineate the cerebral edema range of TBI group and LITUS treatment group on 3D-T2WI layer by layer and the edema volume was obtained after the delineation (Figure 2). In the measurement of T2 edema volume, 2D FLASH image was used as a reference to avoid the area of cerebral hemorrhage (if there was hemorrhage, it would appear as low signal in FLASH image) to prevent the disturbance of hemorrhage on the measurement of signal and edema volume.

The above T2 and volumetric measurements were both performed by two individuals with more than 10 years of neurological MRI diagnosis. Intra-group correlation coefficients (ICC) of the measurements were calculated after the procedure.

### Immunohistochemical Staining

After the last MRI scan (42 days after modeling), the rats were sacrificed under 10% chloral hydrate (4 mL/kg) and the brains collected for HE staining, aquaporin-4 (AQP-4), and β-amyloid precursor protein (β-APP) staining. Prior to histological assessment, the brain tissue was first perfusion fixed as follow procedures: after anesthesia, the rats were fixed on a homemade surgical board, exposed and dissociated from the heart. The perfusion needle was inserted into the left ventricle and fixed, then the right auricle was cut open, and normal saline (4°C) was perfused until the color of the liver and lungs turned white and the outflow fluid of the right atrium was clarified.

#### HE Staining

Rat brains were removed, post-fixed in 4% paraformaldehyde for 48 h. Embedding center (Histo-line Co., Ltd., TEC 2900) was used for paraffin embedding treatment of tissues. Tissues were placed in 60°C paraffin for 3 times, and the time was 30 min, 2 and 1 h, respectively. The paraffin sectionalizer (Leica SM2000R) was then used for continuous section along the coronal position for HE and immunohistochemical staining. Coronal sections were made at the reference to bregma: from −1 to −2 mm and cut on a cryostat (−20°C) at an 5 μm thickness and mounted on slides. The slices were dewaxed in xylene for 5~10 min (19). After the sections were dewaxed by xylene, they were dehydrated with gradient alcohol and stained with hematoxylin dye for 1–2 min. After several seconds of differentiation, the sections were washed with a differentiation solution and after the nuclei appeared blue with the return blue solution for several seconds, washed again. After staining with eosin solution for 10 min, sections were dehydrated with gradient alcohol and sealed with neutral gum. Finally, sections were subjected to microscope observation, photography, and archiving. Each stained section was examined by a research team member with over 10 years of experience using light microscopy. Images were taken using light microscope BX43F, Olympus.

#### AQP-4 and β-APP Staining

The procedure and method of taking brain, making paraffin block and slice are the same as HE staining. First, prepare a methanol solution of 3% hydrogen peroxide, then the sections were dewaxed, and the samples were soaked in a methanol mixture of 3% hydrogen peroxide for 10–15 min. They were washed with PBS 3 times, sealed with normal goat serum working fluid and then incubated 6–8 h in rabbit anti- AQP−4/β–APP antibody (1:400, Chemicon International, Inc., CA). The slides were washed with PBS 3 times followed by incubation in biotinylated anti-rabbit antibody produced in goat (Vector Laboratories, Burlingame, CA) for 1 h. After PBS washes, DAB chromogenic agent (Vector Laboratories, Inc.) was added for 3–5 min. After PBS rinsing, hematoxylin dye was added for several seconds after the specimens were rinsed and dried, they were sealed with neutral resin. Images were taken using light microscope BX43F, Olympus). All brain tissue in the three groups were run up together with the same procedure in order to improve the comparability between different brain tissues of mice and avoid the influence of different DAB concentrations and effects on immunohistochemical images.

#### Average Optical Density (AOD) Analysis

Five high-power fields (×200) of AQP-4 and β-APP staining from each section were randomly selected for image analysis. Image-Pro Plus software (IPP; produced by Media Cybernetics Corporation, USA) has been used in many biological studies for the quantification of proteins formaldehyde (20, 21). In this study, IPP was used to calculate the average optical density. First the analyzed images were opened, Integrated Morphometry selected in the Measure menu, and a comprehensive morphological measurement analysis conducted for the images. Optical density was selected and the positive staining areas were measured to obtain the average optical density value and diameter of all cell staining areas in the image. The value to which the intensity value of each yellow pixel on an image is accumulated is called the integrated optical density (IOD). IOD is then divided by the area of the image to obtain the average optical density (AOD). AOD measured in this study reflects the dyeing density of individual DAB within the unit area (cm^2^) of each image. However, the degree of DAB labeling is indeed closely related to its concentration and reaction time. All tissue was run up together during immunohistochemical staining, namely all samples were stained with the same reagent and under the same conditions. In addition, before AOD measurement, we firstly corrected the image in IPP software, removed the background, and converted the color image into a gray image with 256 gray scales, so as to minimize the impact caused by different dyeing conditions. Two pathologists with more than 10 years of experience performed the measurements and calculated the intra-group correlation coefficients.

### Water Content of Brain Tissue

Rats in each group were subjected to dry and wet weighing, 42 days after TBI. After anesthesia, their heads were cut off and their brains removed. Tissues including cerebrum, cerebellum and brainstem were removed. Brain water content = (wet Weight – dry weight)/wet weight ×100%.

### Neurological Behavioral Scoring

Ambulation, coordination and balance training was conducted for all rats 7 days before modeling. Modified neurological severity score (mNSS) was performed on all rats in the three groups at days 1, 7, 14, 21, 28, 35, and 42 after TBI (22). It includes: (1) tail lift reflection (normal 0 points, max 3 points); (2) walking test (normal 0 points, max 3 points); (3) sensory test (normal 0, max 2); (4) balance test (normal 0 points, max 6 points); (5) lack of reflex and abnormal movement (normal 0, max 4). A lack of a reflex or an animal's inability to complete a task was given a score of 1. Supplementary Table 1 shows a set of modified Neurological Severity Scores (NSS). Neurological function was graded on a scale of 0–18 (normal score, 0; max deficit score, 18). The higher the score, the more severe the brain injury. Two researchers scored the rats' neurological behavior and calculated the intra-group correlation coefficient.

### Statistical Analysis

Statistical analyses were performed using standard software packages (GraphPad Prism version 7.00, La Jolla, CA, USA). Data are presented as the mean ± SD. Data were tested using Shapiro–Wilk tests. The intra-group correlation coefficients of the repeated two-time measurements of T2, volume and neurological behavioral scoring were calculated to verify the consistency of the measurements. For neuroscore and T2 values, groups were compared using the two-way analysis of variance for repeated measures followed by Tukey's *post-hoc* test. For edema volume, TBI and TBI+LITUS groups were compared using Welch's test for its non-normal distribution. For histological and water content analysis, groups were compared using one-way ANOVA. Differences were considered significant at *P* < 0.05.

## Results

### T2 Value in the Damaged Area

**Figure 3:**
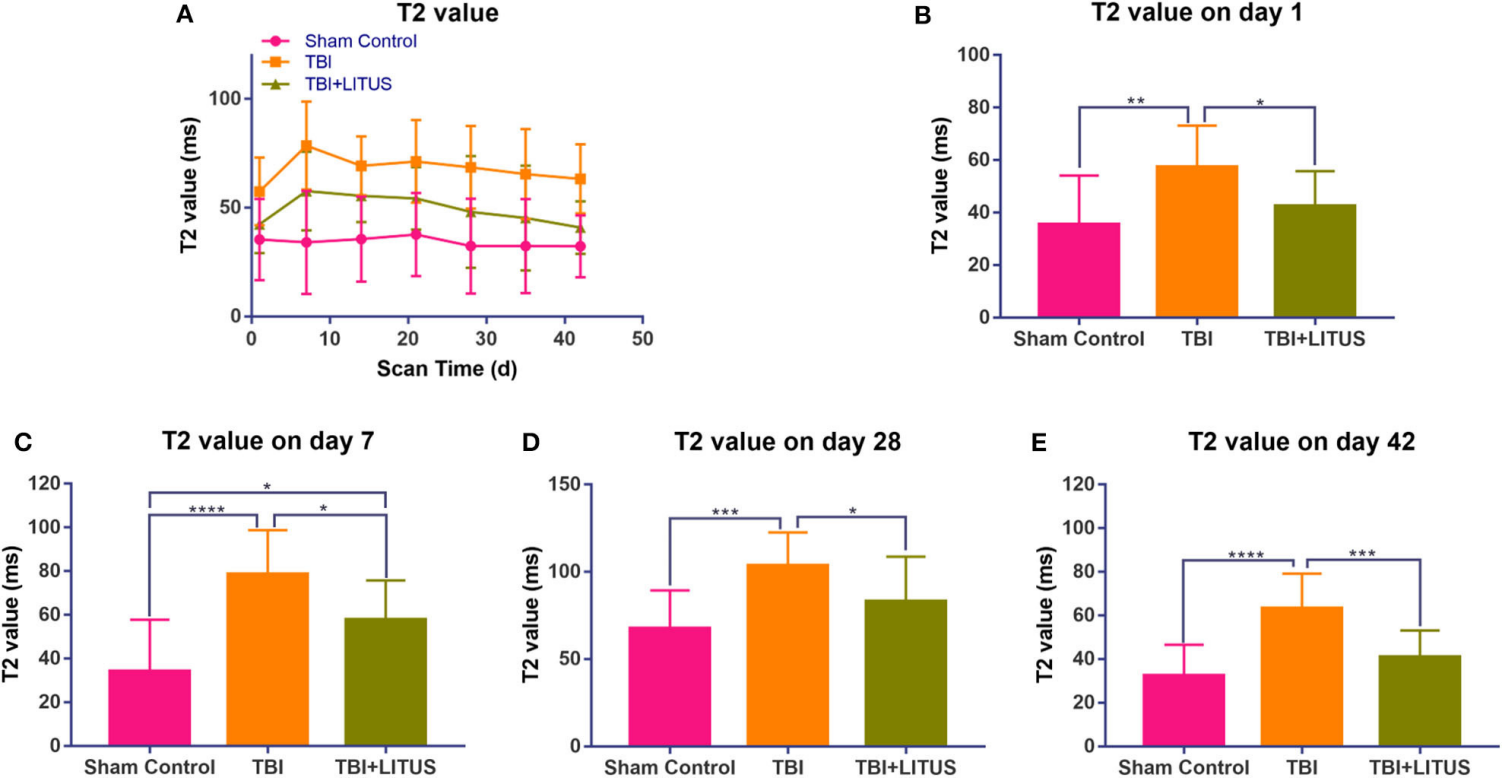
**(A)** Trends of T2 values in the injury cortex. **(B–E)** T2 values on days 1, 7, and 42 in the injury cortex. Data are mean ± SD, *n* = 15. Tukey's *post-hoc* test, *P < 0.05, **P < 0.01, ***P < 0.001, ****P < 0.0001.

TBI increase in T2 signal and then the decrease over time. One day after modeling, T2 values in the TBI group were higher than the sham control group (92.4 ± 15.7 vs. 70.4 ± 18.7, adjusted *P* = 0.0015, *q* = 4.569, degree of freedom = 294, Figure 3 and Supplementary Table 2). The T2 value peaked at day 7 and was significantly higher than the sham control group (113.5 ± 20.2 vs. 69 ± 23.8, adjusted *P* < 0.0001, *q* = 9.241, degree of freedom = 294, Figure 3 and Supplementary Table 2). However, it was still significantly higher than the control group and did not return to the normal levels in the later stage. Although the T2 value in the TBI+LITUS group was significantly higher than the control group, there was a significant difference between the TBI and TBI+LITUS rats at each observation time point. T2 values decreased slowly with continuous treatment for TBI+LITUS group. At day 28, the T2 values were not significantly different from the control group (83 ± 25.7 vs. 67.4 ± 21.9, adjusted *P* = 0.0535, *q* = 3.24, degree of freedom = 294, Figure 3 and Supplementary Table 2), but were significantly lower than the TBI group at day 42 (80.3 ± 24.1 vs. 100.4 ± 20.7, adjusted *P* = 0.0003, *q* = 7.497, degree of freedom = 294, Figure 3 and Supplementary Table 2).

### Volume of Brain Edema

**Figure 4:**
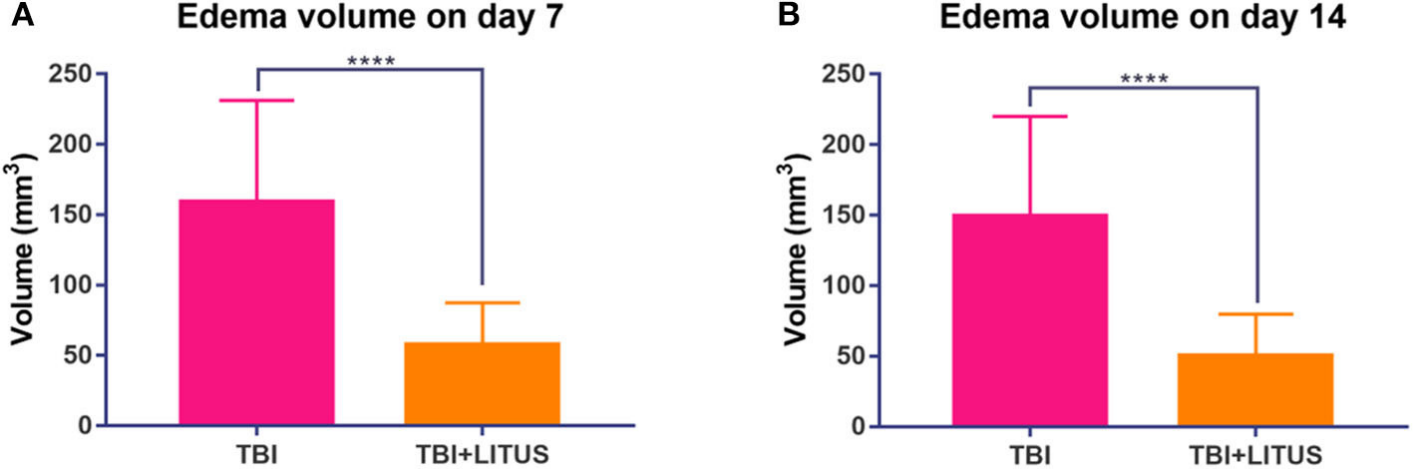
**(A)** Edema volume in injury cortex on day 7. **(B)** Edema volume in injury cortex on day 14. Data are mean ± SD, *n* = 15. Unpaired *t*-test, ****P < 0.0001.

**Figure 5:**
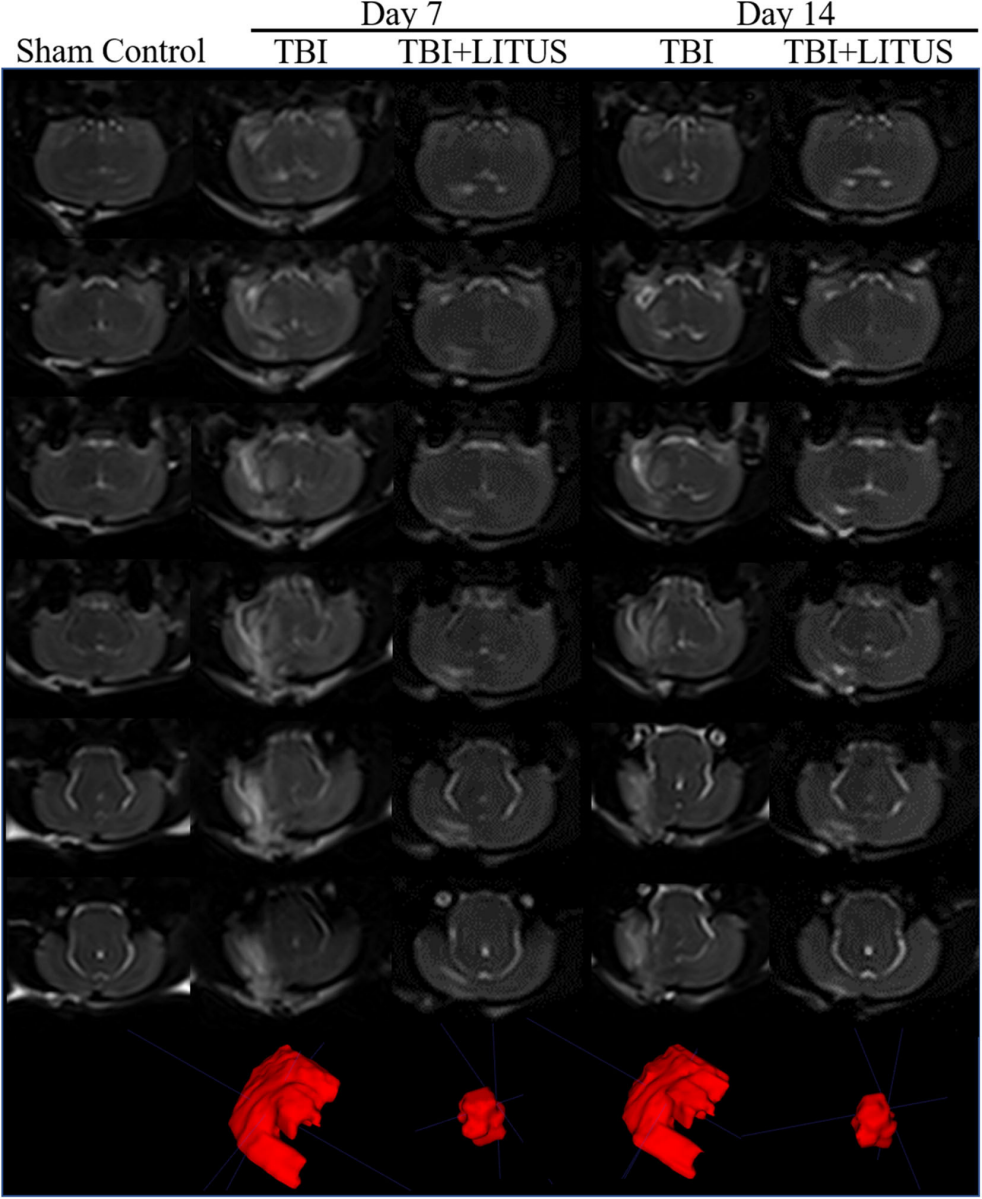
Volume of edema on 3D-T2WI. Representative 3D T2-weighted MRI images at 7 and 14 days post-TBI. The areas with high signals in the figure represent the edema areas in the brain after TBI, and the edema areas of TBI+LITUS are significantly smaller than those in the TBI group (*n* = 15).

Obvious edema area could be observed in the cerebral cortex of the TBI group from day 7 up to 42 days after the trauma and the edema area gradually decreased over time. The edema range in the LITUS treatment group could only be accurately displayed at two time points, days 7 and 14 after the scan, while the boundary of the edema area at other time points was unclear making volume measurement difficult. The edema ranges of the TBI group at 7 and 14 days were about 158.8 ± 72.4 mm^3^ and 149.2 ± 70.6 mm^3^, while those of the LITUS group were about 57.6 ± 29.7 mm^3^ and 50.4 ±29.3 mm^3^. Welch's test showed that the edema volume in these two time points in the LITUS group was significantly lower than the TBI group (both *P* < 0.0001, respectively) (Figure 4 and Supplementary Table 3). The representative T2WI is shown in Figure 5.

### AQP-4 and β-APP Expression in Sham Control, TBI, and TBI+LITUS Group

**Figure 6:**
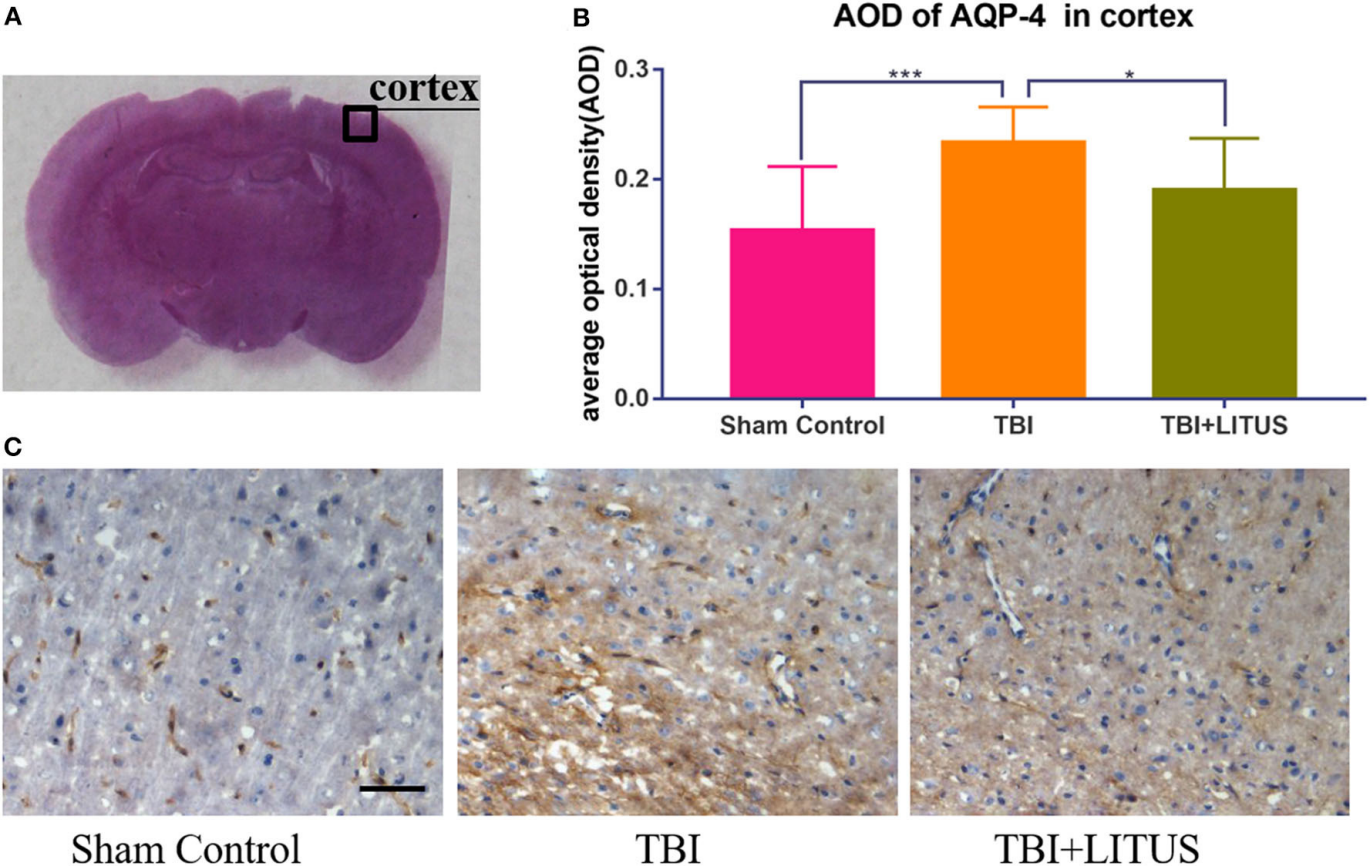
Histopathology in the ipsilateral cortex at 42 days after TBI. **(A)** Representative images of AQP-4 stained neurons at injury cortex. **(B)** The AOD of rats in the TBI+LITUS group was 0.19 ± 0.05. The AOD for the TBI group was 0.23 ± 0.03, indicating LITUS can significantly promote the expression of BDNF AQP-4 staining of the cortex of rat brains of sham control, TBI and TBI+LITUS groups at 42 days after injury. **(C)** Compared to sham control rats, the number of AQP-4 stained cells in the damaged cortex of the TBI group was significantly increased. The number of cell damage in the TBI+LITUS group was significantly less than that in the TBI group, which indicated that LITUS had a good neuroprotective effect. Bar, 50 μm. Data are mean ± SD, *n* = 10. Tukey's *post-hoc* test, *P < 0.05, ***P < 0.001.

**Figure 7:**
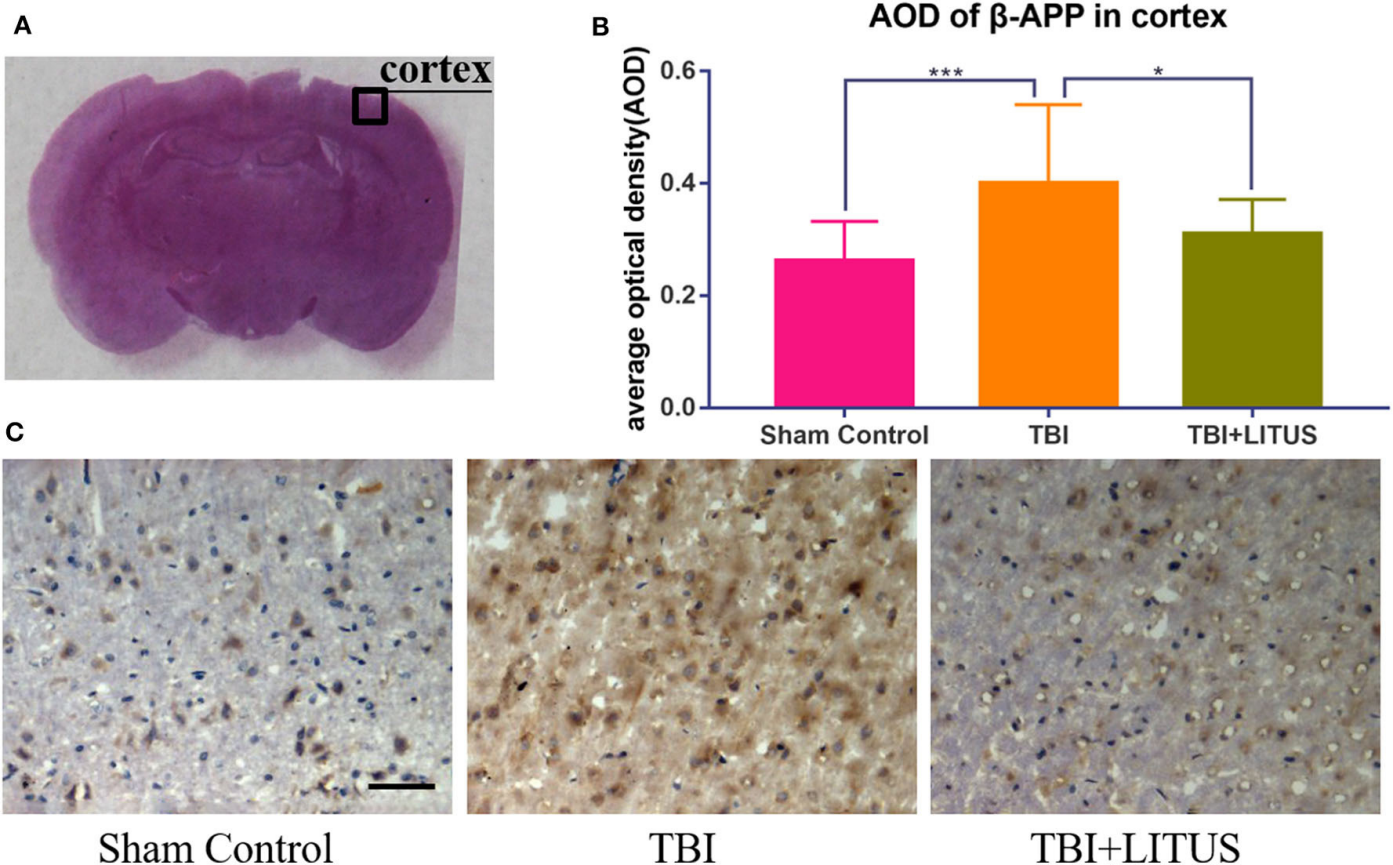
Histopathology in the ipsilateral cortex at 42 days after TBI. **(A)** Representative images of β-APP stained neurons at injury cortex at 42 days after injury. **(B)** The AOD of rats in the TBI+LITUS group was 0.31 ± 0.06. The AOD for the TBI group was 0.4 ± 0.14, indicating LITUS can significantly promote the expression of β-APP. **(C)** Compared to sham control rats, the number of β-APP stained cells in the damaged cortex of the TBI group was significantly increased. The number of cell damage in the TBI+LITUS group was significantly less than that in the TBI group, which indicated that LITUS had a good neuroprotective effect. Bar, 50 μm. Data are mean ± SD, *n* = 10. Tukey's *post-hoc* test, *P < 0.05, ***P < 0.001.

On day 42 after CCI, HE staining showed that neurons in the TBI group had obvious edema, decreased cell density, sparse arrangement, increased cell space, and a light staining of nucleus partial to the center. In addition, the edema area of TBI rats was significantly larger than that of the TBI+LITUS and the sham control group. In the TBI group, AQP-4 and β-APP protein staining showed a strong positive reaction near the CCI point, TBI+LITUS group showed a medium positive reaction, and the sham control group showed a weak positive reaction. Semi-quantitative analysis found that the average optical density of AQP4 in the TBI group, TBI+LITUS group, and sham control group was 0.23 ± 0.03, 0.19 ± 0.05, and 0.15 ± 0.06, respectively (adjusted *P* < 0.0001 for sham control vs. TBI; adjusted *P* = 0.1005 for sham control vs. TBI+LITUS; adjusted *P* = 0.0384 for TBI vs. TBI+LITUS, Figure 6), and the average optical density of β-APP was 0.4 ± 0.14, 0.31 ± 0.06, and 0.26 ± 0.07, respectively (adjusted *P* = 0.0010 for sham control vs. TBI; adjusted *P* = 0.3802 for sham control vs. TBI+LITUS; adjusted *P* = 0.0391 for TBI vs. TBI+LITUS, Figure 7). This indicates that LITUS stimulation had a good effect on the production of harmful proteins.

### Neurological Behavioral Scoring

**Figure 8:**
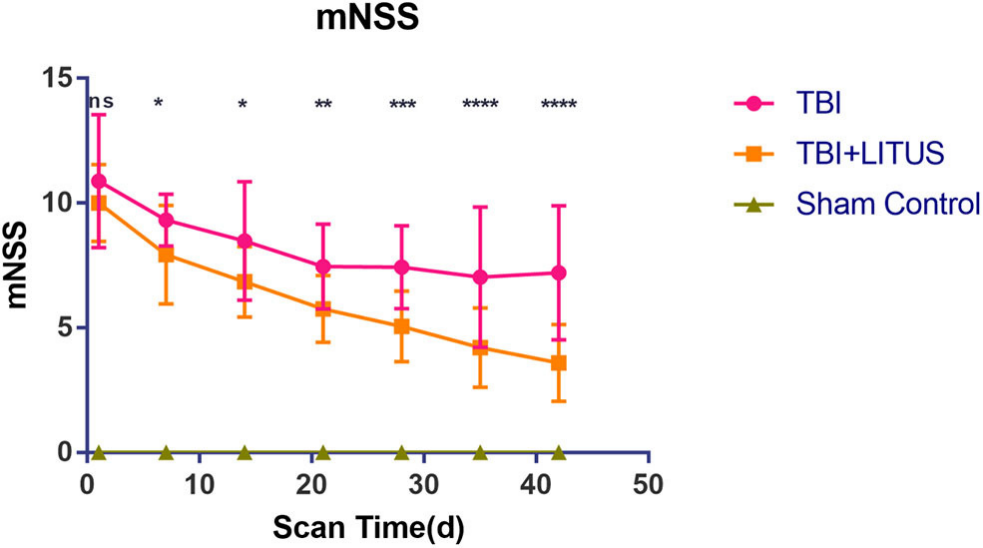
Trends of mNSS of Sham Control, TBI and TBI+LITUS groups. Data are mean±SD, *n* = 15. Tukey's *post-hoc* test for TBI vs. TBI+LITUS, *P < 0.05, **P < 0.01, ***P < 0.001, ****P < 0.0001.

**Figure 9:**
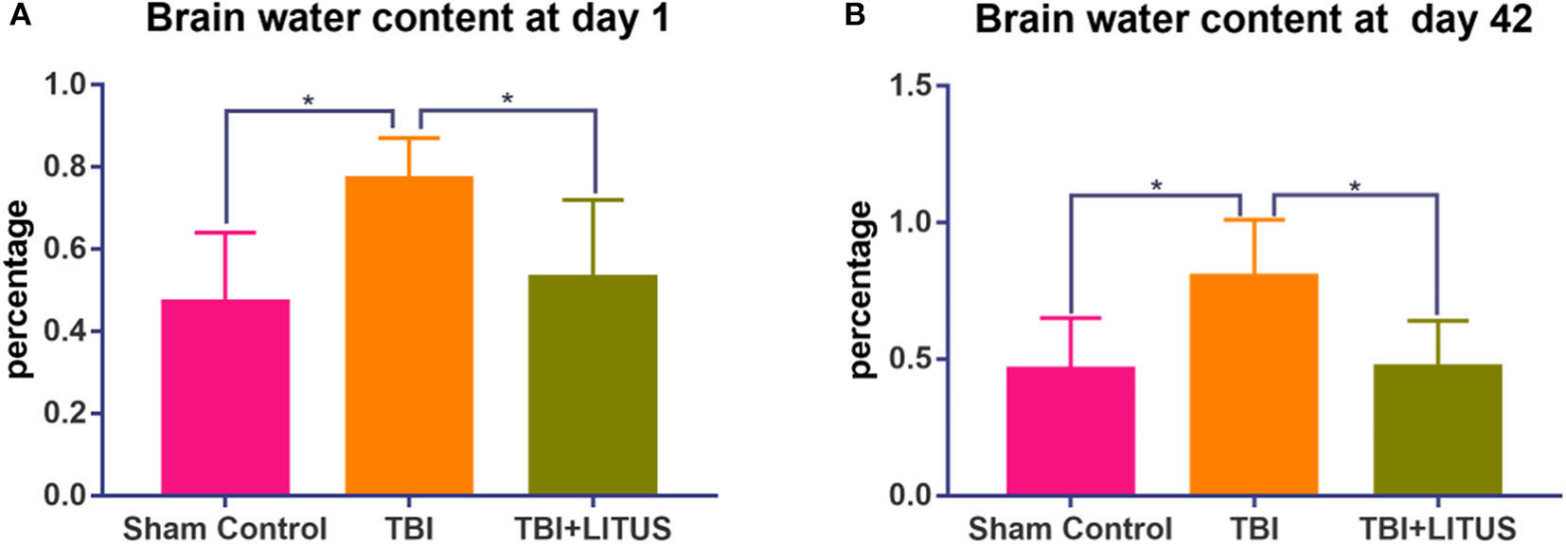
Water content of TBI and TBI+LITUS groups. **(A)** Brain water content at day 1. **(B)** Brain water content at day 42. Data are mean ± SD, *n* = 5, respectively, for the two time points. Tukey's *post-hoc* test for TBI vs. TBI+LITUS. Unpaired *t*-test, *P < 0.05.

For TBI and TBI+LITUS, the mNSS score decreased gradually over time. The variation ranges were 10.88 ± 2.66 to 7.2 ± 2.69 and 10.1 ± 1.54 to 3.59 ± 1.54, respectively. The scores of the two groups were similar on day 1, however, the range of the TBI+LITUS group was larger, indicating a significant recovery of rats' neural function in the TBI+LITUS group. Except for 1 day after injury, there were significant differences in scores among groups at each subsequent time point (adjusted *P* = 0.0415, 0.0123, 0.0090, 0.0001, < 0.0001, < 0.0001 for days 7, 14, 21, 28, 35, and 42, Figure 8 and Supplementary Table 4). Through the curve, we found that the mNSS of the rats in the TBI group did not recover significantly after 21 days of exhibiting a plateau change, while the scores of the rats in the TBI+LITUS group had a continuous downward trend. In addition, there was an interaction between time factor and LITUS treatment (Greenhouse-Geisser, *F* = 223.34, *P* < 0.0001), that is, the effect of time factor on TBI and LITUS group was slightly different. It was shown in Figure 9 that the mNSS value of LITUS group decreased more rapidly with time, that is, LITUS improved the Neurological function of rats more and more significantly with the prolongization of treatment time.

### Water Content

The brain water content in the TBI group increased significantly compared with that in the control group at the early and later stage of the trauma (0.77 ± 0.10 vs. 0.47 ± 0.17, adjusted *P* = 0.0277, 1 day after TBI; 0.80 ± 0.21 vs. 0.46 ± 0.19, adjusted *P* =0.0383, 42 days after TBI, Figure 9). In the TBI+LITUS group, the water content in the brain was significantly reduced compared to the TBI rats (0.50 ± 0.19 vs. 0.77 ± 0.10, adjusted *P* =0.0473, 1 day after TBI; 0.47 ± 0.17 vs. 0.80 ± 0.21, adjusted *P* =0.0443, 42 days after TBI, Figure 9). These results suggest that LITUS stimulation can significantly reduce cerebral edema at both early and later stage.

## Discussion

T2WI is a common MRI sequence used in the clinical evaluation of various brain edemas. Brain is defined as local accumulation of water molecules and prolonged T2 relaxation time. Therefore, a brain edema area shows a high signal which is significantly different from the normal brain tissue in the T2WI. Therefore, T2WI can be used as a sensitive sequence for studying the range and degree of brain edema. In this study, iso-voxel 3D-T2WI sequence was used to maintain the isotropy of the image under the premise of thinner layers, thus ensuring the accuracy of the brain edema volume measurements. In addition, we used multi-TE T2WI to dynamically measure the T2 value in the edema area. Currently, T2 mapping is widely used for various systems of the body, most commonly for lumbar annulus degeneration, articular cartilage injury in limbs and ischemia, and inflammation in the heart (23–25). In recent years, researchers have used it alone or in combination with other techniques in order to evaluate brain injury caused by trauma, ischemia, and developmental disorders (26, 27). T2 mapping is highly sensitive to cerebral edema and the severity of edema can be observed with a new perspective by breaking down the limitation of the edema volume measurement, and in turn, provide us with a highly sensitive quantitative index for LITUS while studying TBI rat edema recovery.

A previous study by a different group already looked at edema with LITUS at 148 days post-CCI in mice (13, 14). And another study looked at LITUS's effects on acute edema (1 and 4 days post-CCI) using MRI and brain weight as well as molecular/pathological targets for this change (13). Compared with these two important studies, our study has the following differences. First, previous studies have measured only the maximum area of edema. In the present study, the T2 value and volume of brain injury was measured to comprehensively reflect the edema of brain tissue. Furthermore, the time span of this study is longer. Through dynamic observation of MR, we not only found that the degree of brain edema was the most severe 7 days after brain trauma, but also found that LITUS treatment could well-inhibit the changes in T2 value and volume of tissues caused by brain edema. The wider time span allows better observation of the dynamic changes in the range of cerebral edema. In addition, the previous studies have found that there is zonula infiltration, neutrophil infiltration, and microglial activation in local lesions in the early stage after brain trauma. Their studies have explored the development mechanism of brain edema mainly from two perspectives of BBB destruction and brain tissue inflammatory cells. In this study, through semi-quantitative analysis of AQP-4 and β-APP protein expression, it was found that increased expression of aquaporin and neuronal injury may also be the mechanism of brain edema.

We found that the T2 value in the injury area of the rats in the treatment group increased slightly at day 1, peaked at day 7, and increased significantly during the observation period compared to the sham control group (Figure 3). Our previous DTI study found that the MD value of damaged brain tissue decreased at 1 day, followed by a gradual increase over the observation period of 7–42 days. In addition to the MD value, diffusion-related parameters such as FA and MK were measured in the damaged brain tissue in our previous studies, and it was found that the local lesion showed limited diffusivity 1 day after the injury, manifested as increased FA and MK, and the MK value peaked at 7 days. After 7 days, FA and MK values decreased gradually with time, indicating that the degree of diffusion limitation decreased gradually (15, 16). The decreased diffusivity is consistent with lowered T2 values in the present study. Therefore, by combining the previous studies, we found that the temporal consistency of the changes of relevant parameters in different imaging methods could confirm the development law of brain tissue injury after TBI from multiple perspectives, especially the discovery that the brain injury was the most severe at 7 days. In addition, our previous studies mainly elucidate the role and mechanism of LITUS on neuronal injury and neuronal microenvironment from the perspective of imaging and pathology. This study mainly analyzed the efficacy of LITUS from another perspective, namely, post-traumatic brain edema and LITUS inhibition of brain edema, and further improved the possible mechanism of LITUS in promoting cerebral trauma recovery.

In this study, the T2 value of the normal group of rats was around 70 ms. Aradi et al. (28) found that the T2 value of the cortex of normal rat was about 75 ± 2 ms in 3T MR study, which was close to the results of our study, indicating that the T2 value from FSE sequence was stable in the same field intensity MR machine, which provided a technical basis for the study of brain trauma and other diseases. Kharatishvili et al. (29) found a positive correlation between the T2 value measured on T2WI images and injury cortical volume on 3 days after TBI with the actual damage volume being confirmed using immunohistochemistry. Quantitative T2 mapping is, therefore, a potential marker for initial assessment of the severity of TBI damage in rats. A previous human clinical study have shown that changes in T2 values are sensitive to edema in brain tissue (27). However, an interesting phenomenon was found in this study, that is, although T2 value increased at the initial stage (day 1) after brain trauma, it was not very significant (Figure 3). Former researchers have furtherly concluded that the increase of T2 value in the brain tissue is mainly due to the water content in brain tissue. After injury, the total water content in the acute phase of pathological measurement increases by about 3–5% and only a slight T2 increase is observed in the brain of TBI model rats (27, 30), consistent with the results of this study. We also found that the T2 value reached its peak at 7 days after injury. Previous studies have confirmed that the water content of reperfusion after injury can increase by up to 28% and lead to a significant increase in T2 value (31). This theory of reperfusion explains our findings very well. However, other researchers have argued that the absolute amount of water is not the only factor that affects T2. T2 relaxation time of free water is around 40 times that of the bound water and the T2 increase reflects the water and protein separation. Previous studies have shown that increase in free water and a decrease in bound water have a greater effect on T2 than changes in the total water content (32). Moreover, damage to brain tissue will lead to the rupture of damaged neurons, which will cause the binding water in the protein tissue to become free water, leading to a significant increase in T2 value (32).

Through volume measurement on iso-voxel 3D-T2WI images, we found significant edema in the localized brain tissue at 7 and 14 days after the CCI induced TBI. At the beginning and end of the study, using the dry and wet weight method, we verified that the brain water content of rats in the TBI group was significantly higher than the control group. Although the striking site of CCI method used in this study was the cerebral cortex, it was observed that the range of brain edema after TBI was not just limited to the cortex, but also extended to the cortical and subcortical white matter regions. The reason may be due to the structural differences between the gray and white matter (33). The structure of white matter is loose, while the structure of gray matter is tight as it is full of neurons, cell bodies, and dendrites (34). Once the gray matter edema infiltrates into the white matter, the edema quickly reaches a wider area and leads to extensive changes of cerebral edema in TBI rats. There are also other possibilities for the difference in gray and white matter edema readings on the T2WI MRI, such as injury localization, blood–brain barrier (BBB) breakdown, white matter concentration and differences in morphology/function of glia (33, 35–37). In addition, some researchers believe that the white matter damage can be caused by the primary insult or the cascade of secondary injuries due to electrolyte imbalances, energy failure, and inflammation (9, 10). The mechanism of white matter damage after TBI, therefore, may be multifaceted.

Although we measured T2 values at all-time points, we measured volumes at only 7 and 14 days. The reason is that the FSE-T2WI sequence adopted in this study can clearly show the boundary of brain edema of rats in TBI+LITUS group only at these two time points, and the edge cannot be accurately delineated at 1 day and 21–42 days of TBI. We speculated that the blurring of the boundary of cerebral edema in LITUS group rats 1 day after TBI may be due to the dominance of cytotoxic edema in the early stage after injury. At this time, the sodium pump was not functioning properly, and the extracellular space water molecules entered the cell, but the local water molecules were not significantly increased, so it was difficult to distinguish the edema area from the surrounding brain tissue by conventional T2WI. This manifestation also suggests that LITUS has a therapeutic effect at the initial stage of application, which has also been confirmed by MR observations in a previous study (13). At 7 and 14 days, the cerebral edema was gradually aggravated and the boundary of edema became clear due to the development of vasogenic edema and intracerebral reperfusion (31, 38). At the time point of 21 days and later, with the cumulative effect of LITUS treatment, brain edema was reduced and T2 value decreased, thus blurry boundary appeared again. Therefore, Compared with T2 volume measurement, T2 value may be more advantageous in the evaluation of cerebral edema after LITUS treatment.

In this study, LITUS was used to continuously stimulate the rat brain after TBI. We found that LITUS can significantly reduce the T2 value of brain injury area, reduce the volume, and shorten the duration of edema. Especially at the end of observation (day 42), LITUS efficiently promoted the decrease of T2 value. For the edema volume, the LITUS treatment group was able to distinguish the edema boundary only at days 7 and 14, and the edema range was significantly smaller than the injury group, indicating that LITUS treatment significantly promoted the reduction of edema. Due to the edema recovery at other time points, the boundary between the damaged area and the non-damaged area is not quite clear and it is difficult to show its exact range. We found that compared to commonly used 3D-T2WI volume measurements, T2 mapping measurement was not affected by the fuzzy edges of the edema as it did not need to determine the edge of edema. Therefore, T2 mapping measurements may be more sensitive to edema than volumetric measurements. This has also been confirmed by Irina Kharatishvili et al. (29) where they found that T2 value can distinguish the severity of brain injury and is correlated with the behavioral prognosis of rats, therefore being more advantageous than volume measurement. At day 42, the reduction in brain water content by the dry and wet weight method confirmed LITUS's effect on the brain edema. In addition to the MRI and brain water content measurements, through continuous neurological score observation, we found that LITUS can promote the rehabilitation of neural function after TBI, indicating that LITUS can improve the behavioral prognosis of TBI rats.

As for the mechanisms of LITUS in cerebral edema treatment, researchers have mainly hypotheses on two aspects, maintaining the integrity of the BBB and promoting the opening of cell ion channels (39, 40). They believe that the destruction of the blood-brain barrier caused by the early stage of TBI increases the capillary permeability, leads to water leakage into the perivascular and intercellular stroma, and manifests as a vasogenic cerebral edema. Meanwhile, LITUS stimulation can protect the activation of endothelial cell 3-kinase/threonine-protein kinase (PI3K/Akt) pathway by increasing the expression of tight junction protein zonula occludens-1 (ZO-1), thus maintaining the integrity of the BBB and reduce BBB dysfunction of commonly observed in TBI (13). Some researchers also pointed out that intracellular toxic edema caused by various pathogenic factors such as the imbalance of the internal and external environment of cells, disturbance of the exchange of cellular Na^+^ –K^+^, and disorder of adenosine triphosphate (ATP) production, mainly manifested due to the accumulation of intracellular sodium and water. LITUS stimulation can lead to structural changes in the lipid biomolecular layer at the local fatty acid transmembrane position and promote the opening or closing of numerous mechanically sensitive ion channels on the membrane, in order to regulate Na^+^ –k^+^ pump function and inhibit cytotoxic edema (40). By observing the T2 value, we found that LITUS had an inhibitory effect on the early and late cerebral edema. Therefore, LITUS may have a certain regulatory effect on both the angiogenic and cytotoxic edema.

In addition to the above regulatory mechanisms, this study suggests that inhibiting the expression of AQP-4 and promoting β-app clearance may also be an important mechanism for LITUS. However, these results were obtained by semi-quantitative methods of immunohistochemistry, and need to be furtherly verified by quantitative methods such as western blot. In this study, we observed that the expression of AQP-4 in the brain tissue increased at the end of the study. After trauma, AQP-4 is expressed in both neurons and glial cells (41). LITUS stimulation can significantly inhibit the expression of AQP-4 in the damaged areas and regulate the occurrence and development of cerebral edema after a TBI. AQP-4, as a transmembrane water transporter, is involved in regulating the balance of water inside the cells. Several studies have confirmed that there is a time-history correlation between the expression of AQP-4 and changes in the brain water content (42, 43). When the expression of AQP-4 increases, the brain water content also increases and edema worsens. Edema causes difficulties for the cells to adjust their volume, resulting in high pressure inside the cell and eventual rupture (44). Therefore, although this study could not accurately distinguish between the two types of cells, it could also make a preliminary judgment on the therapeutic effect of LITUS.

In addition, we found that β-APP increased significantly in the white matter of the damaged brain while LITUS inhibited the expression of β-APP. β-APP is a large molecular membrane glycoprotein with a receptor-like structure, whose expression levels in normal brain tissue is quite low and therefore difficult to detect. Under mechanical damage, axon transport obstacles occur after axon damage, resulting in abnormal aggregation and up-regulated expression of β-APP at the damaged site. β-APP is often used as a marker for the detecting of axonal damage. Smith et al. showed that damaged nerve axons release large amounts of β-APP into the surrounding brain parenchyma (45). Ekmark et al. (46) also found that β-APP content in the brain tissues increase significantly after diffuse axonal injury (DAI). Some researchers found that LITUS can reduce the accumulation of harmful substances in neurodegenerative diseases. Previous studies have also shown that LITUS may indirectly inhibit brain edema by improving axoplasmic transport barriers (47). In addition, prevention of neuronal and axonal injury may also be another reason for LITUS to reduce APP expression and combat brain edema. Our previous studies have found that LITUS can inhibit the abnormal diffusion related parameters in local injury or even distant brain tissue after TBI, and these parameters are closely related to the structural abnormalities of axonal nerve, indicating that LITUS treatment can reduce in overall axonal injury (15, 16). There are even studies showing that LITUS can promote axonal regeneration by stimulating the production of neuroprotective factors such as BDNF (48, 49). Therefore, promoting the removal of harmful substances such as β-APP and inhibition of neuroaxonal injury may be an important mechanisms of LITUS in anti-traumatic cerebral edema.

This study also scored the changes in the neural function of rats during the whole experimental period. The normal rats were rated as 0. At the early stage of brain trauma, the nerve score was the highest, indicating the most serious neuron damage. LITUS treatment effect had not been reflected at day 1, so there was no significant difference in the score between LITUS group and TBI group. As time goes on, the difference between the two groups becomes increasingly significant. The recovery rate of neurological function in LITUS group was significantly faster than that in TBI group. The results of ANOVA of repeated measurements also showed that there was an interaction between time factors and treatment factors. Combined with the Figure 8, we speculated that the accumulation of treatment time could improve the treatment effect of LITUS. Some previous studies on TBI have also found that LITUS can not only improve the prognosis of TBI in microscopic histology, but also improve its behavioral prognosis in macroscopic perspective (13, 14). Therefore, although LITUS may not be able to change the neurological function score of rats 1 day after injury, sustained LITUS treatment significantly improves the behavioral prognosis of rats.

This study has several limitations. First, the sequence used in this study to obtain T2 value is a TSE sequence. When scanning, applying a 180-degree pulse causes a lot of heat to be generated inside a human body, especially in ultra-high field magnetic resonance equipment. Second, this study manually outlined ROI in order to measure the T2 value of the damaged area. For different scanning individuals, the ROI may be different. Third, when conducting volume measurement, this study adopted the method of layer by layer measurement followed by automatic superposition of the ITK-SNAP software, which may be subjective when sketching. Fourth, Co-localization studies subject to experimental conditions were not performed to clarify neuron vs. astrocyte staining of AQP-4. In addition, when AOD method was used to conduct semi-quantitative research on protein expression, factors such as DAB solution concentration were still one of the important factors influencing the results, although we made staining conditions of tissues as much as possible and made correction in advance during image evaluation.

In conclusion, this study demonstrated that the LITUS once a day for 6 weeks has a positive therapeutic effect on post-traumatic brain edema confirmed through 3D-T2WI and multi-TE imaging, and the potential mechanism may be related to the inhibition of the expression of AQP-4 edema promoting substances and promotion of β-APP. These findings suggest that LITUS treatment may be useful for preventing traumatic brain edema and serve as a promising new strategy for clinical use in the future.

## Data Availability Statement

The original contributions presented in the study are included in the article/Supplementary Materials, further inquiries can be directed to the corresponding author/s.

## Ethics Statement

The animal study was reviewed and approved by Qinhuangdao Municipal No. 1 Hospital.

## Author Contributions

TZ, YY, YJ, and LL designed and coordinated the study. TZ, JD, XW, and QS carried out experiment and data process, and drafted the manuscript. All authors gave final approval for publication.

## Conflict of Interest

QS was employed by the company Siemens Ltd. The remaining authors declare that the research was conducted in the absence of any commercial or financial relationships that could be construed as a potential conflict of interest.
