# Supplementary material for: Effect of Low Intensity Transcranial Ultrasound (LITUS) on Post-traumatic Brain Edema in Rats: Evaluation by Isotropic 3-Dimensional T2 and Multi-TE T2 Weighted MRI
Source: Front Neurol. 2020 Nov 12;11:578638. doi: 10.3389/fneur.2020.578638 (PMC7689022; doi:10.3389/fneur.2020.578638)
Supplement: Supplementary file 1 [file Table_1.DOCX]

**Supplement Materials**

**Supplement TABLE 1. Modified Neurological Severity Scores (NSS)**

|  | Points |
| --- | --- |
| Motor tests |  |
| Raising rat by the tail | 3 |
| 1 Flexion of forelimb |  |
| 1 Flexion of hindlimb |  |
| 1 Head moved >10° to vertical axis within 30 s |  |
| Placing rat on the floor (normal=0; maximum=3) | 3 |
| 0 Normal walk |  |
| 1 Inability to walk straight |  |
| 2 Circling toward the paretic side |  |
| 3 Fall down to the paretic side |  |
| Sensory tests | 2 |
| 1 Placing test (visual and tactile test) |  |
| 2 Proprioceptive test (deep sensation, pushing the paw against the table edge to stimulate limb muscles) |  |
| Beam balance tests (normal=0; maximum=6) | 6 |
| 0 Balances with steady posture |  |
| 1 Grasps side of beam |  |
| 2 Hugs the beam and one limb falls down from the beam |  |
| 3 Hugs the beam and two limbs fall down from the beam, or spins on beam (>60 s) |  |
| 4 Attempts to balance on the beam but falls off (>40 s) |  |
| 5 Attempts to balance on the beam but falls off (>20 s) |  |
| 6 Falls off: No attempt to balance or hang on to the beam (>20 s) |  |
| Reflexes absent and abnormal movements | 4 |
| 1 Pinna reflex (head shake when touching the auditory meatus) |  |
| 1 Corneal reflex (eye blink when lightly touching the cornea with cotton) |  |
| 1 Startle reflex (motor response to a brief noise from snapping a clipboard  paper) |  |
| 1 Seizures, myoclonus, myodystony |  |
| Maximum points | 18 |

One point is awarded for the inability to perform the tasks or for the lack of a tested reflex; 13 to 18 indicates severe injury; 7 to 12, moderate injury; 1 to 6, mild injury.

**Supplement table 2. T2 values at the focal lesion of the Sham Control (SC), TBI and TBI+LITUS groups**

|  | SC VS. TBI（n=15） | Adjusted P | SC VS. TBI+LITUS（n=15） | Adjusted P | TBI VS. TBI+LITUS（n=15） | Adjusted P |
| --- | --- | --- | --- | --- | --- | --- |
| T2 at day 1 | 70.4±18.7 VS. 92.4±15.7 | 0.0015^**^ | 70.4±18.7 VS. 77.4±13.4 | 0.4644 | 92.4±15.7 VS. 77.4±13.4 | 0.0374^*^ |
|  |  |  |  |  |  |  |
| T2 at day 7 | 69±23.8 VS. 113.5±20.2 | <0.0001^****^ | 69±23.8 VS. 92.6±18.2 | 0.0095^**^ | 113.5±20.2 VS. 92.6±18.2 | 0.0237^*^ |
|  |  |  |  |  |  |  |
| T2 at day 14 | 70.5±19.5 VS. 104.2±13.6 | <0.0001^****^ | 70.5±19.5 VS. 90.4±12.1 | 0.0028^**^ | 104.2±13.6 VS. 90.4±12.1 | 0.0473^*^ |
|  |  |  |  |  |  |  |
| T2 at day 21 | 72.6±19.1 VS. 106.2±19 | <0.0001^****^ | 72.6±19.1 VS. 89.3±14.4 | 0.0341^*^ | 106.2±19 VS. 89.3±14.4 | 0.0317^*^ |
|  |  |  |  |  |  |  |
| T2 at day 28 | 67.4±21.9 VS. 103.5±19 | 0.0002^***^ | 67.4±21.9 VS. 83±25.7 | 0.1484 | 103.5±19 VS. 83±25.7 | 0.0415^*^ |
|  |  |  |  |  |  |  |
| T2 at day 35 | 67.4±21.6 VS. 100.4±20.7 | 0.0006^***^ | 67.4±21.6 VS. 80.3±24.1 | 0.2599 | 100.4±20.7 VS. 80.3±24.1 | 0.0443^*^ |
|  |  |  |  |  |  |  |
| T2 at day 42 | 67.3±14.2 VS. 98.2±15.9 | <0.0001^****^ | 67.3±14.2 VS. 75.9±12.1 | 0.2308 | 98.2±15.9 VS. 75.9±12.1 | 0.0003^***^ |
|  |  |  |  |  |  |  |
| Values are represented as mean±SD. | | | | | | |

**Supplement table 3. Edema volume at the focal lesion of the TBI and TBI+LITUS groups**

|  | TBI VS. TBI+LITUS（n=5） | Adjusted P |
| --- | --- | --- |
| Volume at day 7 | 158.8±72.4 VS. 57.6±29.7 | <0.0001^*^ |
| Volume at day 14 | 149.2±70.6 VS. 50.4±29.3 | <0.0001^*^ |
| Values are represented as mean±SD. | | |

**Supplement table 4. mNSS scores of the TBI and TBI+LITUS groups**

|  | TBI VS. TBI+LITUS（n=15） | Adjusted P |
| --- | --- | --- |
| day 1 | 10.88±2.66 VS. 10.01±1.54 | 0.2966 |
| day 7 | 9.32±1.04 VS. 7.93±1.98 | <0.0001^*^ |
| day 14 | 8.48±2.37 VS. 6.84±1.41 | <0.0001^*^ |
| day 21 | 7.45±1.71 VS. 5.75±1.34 | <0.0001^*^ |
| day 28 | 7.43±1.66 VS. 5.06±1.41 | <0.0001^*^ |
| day 35 | 7.03±2.81 VS. 4.2±1.59 | <0.0001^*^ |
| day 42 | 7.2±2.69 VS. 3.59±1.54 | <0.0001^*^ |
| Values are represented as mean±SD. | | |
